# *Escherichia coli* challenge and one type of smectite alter intestinal barrier of pigs

**DOI:** 10.1186/2049-1891-4-52

**Published:** 2013-12-20

**Authors:** Juliana Abranches Soares Almeida, Yanhong Liu, Minho Song, Jeong Jae Lee, H Rex Gaskins, Carol Wolfgang Maddox, Orlando Osuna, James Eugene Pettigrew

**Affiliations:** 1 Department of Animal Sciences, University of Illinois, Urbana 61801, USA; 2 Current address: Department of Animal Science and Biotechnology, Chungnam National University, Daejeon, South Korea; 3 Department of Pathobiology, University of Illinois, Urbana 61801, USA; 4 Milwhite, Inc., Brownsville, TX, USA

**Keywords:** Barrier function, *E. coli*, Pigs, Smectite, Zeolite

## Abstract

An experiment was conducted to determine how an *E. coli* challenge and dietary clays affect the intestinal barrier of pigs. Two groups of 32 pigs (initial BW: 6.9 ± 1.0 kg) were distributed in a 2 × 4 factorial arrangement of a randomized complete block design (2 challenge treatments: sham or *E. coli*, and 4 dietary treatments: control, 0.3% smectite A, 0.3% smectite B and 0.3% zeolite), with 8 replicates total. Diarrhea score, growth performance, goblet cell size and number, bacterial translocation from intestinal lumen to lymph nodes, intestinal morphology, and relative amounts of sulfo and sialo mucins were measured. The *E. coli* challenge reduced performance, increased goblet cell size and number in the ileum, increased bacterial translocation from the intestinal lumen to the lymph nodes, and increased ileal crypt depth. One of the clays (smectite A) tended to increase goblet cell size in ileum, which may indicate enhanced protection. In conclusion, *E. coli* infection degrades intestinal barrier integrity but smectite A may enhance it.

## Background

Weaning is a stressful period for piglets due to environmental, social and nutritional changes. During this period, pigs are also vulnerable because of their immature immune and digestive systems [1]. The stress may result in depressed feed intake which may lead to poor performance and changes in the intestinal structure and microbiota, thus increasing the susceptibility of pigs to enteric diseases [2]. Post-weaning diarrhea caused by *Escherichia coli* is a common enteric disease in weaned pigs; it causes economic losses due to mortality, morbidity, decreased growth performance and cost of medication [3]. Diarrhea also impairs nutrient absorption, increases permeability in the intestine, decreases tight junction integrity, increases paracellular movements of molecules and increases infection [4]. Among a large number of potential mechanisms are mucosal injury, villous atrophy, increased mast cell number, and reduction in numbers of lymphocytes subsets (CD8^+^ T and CD4^+^ T) in jejunum and ileum [4,5].

Antibiotics suppress growth of certain microorganisms and are widely used as growth promoters in the swine industry [6]. However, concern over their potential contribution to antibiotic resistance in bacteria infecting humans has led to tightening restrictions on antibiotic use in animals, including cessation of their use as growth promoters in Denmark in May 1995 [7] and elsewhere more recently. The resulting reduction of growth performance and increase in the morbidity in nursery pigs in Denmark indicate the need for prophylaxis [7]. Therefore, it is important to find other reliable strategies to maintain pig health. Among several alternatives, clays have shown promise [8].

Clays have been used in human medicine to ameliorate diarrhea [9], and they are also used in the pig industry with some success [8,10,11]. In the livestock industry, clays are used mainly as mycotoxin binders and as additives that contribute to improve the flow of the feed in bins and feeders, reducing problems with caking of feed. Clays have not been shown to consistently alter growth performance [12-14]. Several types of clays are available and they appear to have different applications and modes of action. Clays with both the 1:1 layer structure (e.g. kaolinite) and the 2:1 layer structure (e.g. smectite) have positive effects on gastrointestinal health of the animals [15,16]. Song et al. reported [8] that, when pigs were challenged with a pathogenic *E. coli,* feeding dietary clays including smectite, zeolite, kaolinite or combinations of them at 0.3% of the diet reduced diarrhea. Thus, the effect of clays on gastrointestinal health seems more consistent and beneficial than the effect of clays on performance.

Knowledge of the mechanisms through which clays specifically improve gastrointestinal health is lacking, but there are indications [15,16] that clays may strengthen the mucus layer of the intestinal barrier. Moreover, the effects of a challenge with a pathogenic *E. coli* on bacterial translocation from intestinal lumen to mesenteric lymph nodes and goblet cell size and number in weaned pigs has not yet been reported. Our objectives were to determine the effects of a pathogenic *E. coli* challenge and of dietary clays on the intestinal barrier of pigs.

## Materials and methods

The Institute of Animal Care and Use Committee of the University of Illinois reviewed and approved the animal care procedures for this experiment.

### Animals, experimental design and diets

Two groups of 32 weanling pigs each (about 21d old; initial BW: 6.9 ± 1.0 kg) were obtained from the Swine Research Center of the University of Illinois. Pigs were housed in disease-containment chambers of the Edward R. Madigan Laboratory building at the University of Illinois at Urbana-Champaign from weaning to about 35 d of age. Pigs had 6 d of adaptation period before challenge. There were a total of 32 individual pens, 4 in each of 8 chambers in each suite. There were 2 suites that were used for either challenged or unchallenged pigs and in each suite, 4 chambers in each suite were used. The treatments were arranged in a 2 × 4 factorial design [without or with *E. coli* challenge and 4 dietary treatments: control, and 0.3% of 3 different clays added to the control diet: smectite A (**SMA**), smectite B (**SMB**) and zeolite (**ZEO**)]. The enterotoxigenic (**ETEC**) *E. coli* used for the challenge was isolated from a field disease outbreak, (isolate number UI-VDL 05–27242). It is an F-18 fimbria + *E. coli* strain that produces the heat-labile toxin, heat-stable toxin b, and Shiga-like toxin-2 [17]. The pigs were orally inoculated with *E. coli* (10^10^ cfu per 3 mL dose) in PBS daily for 3 d continuously to cause mild diarrhea [17]. The unchallenged treatment (sham) received a 3 mL dose of PBS daily for 3 d. Both inoculations were given orally beginning 6 d after weaning (d 0). Personnel conducting the experiment were blind to the dietary treatments.

The complex nursery basal diet [8] was formulated to meet or exceed NRC [18] estimates of requirements of weanling pigs (Table 1). All the other experimental diets were made from the basal and the addition of 0.3% of each dietary clay. It did not include spray-dried plasma, antibiotics, or zinc oxide to avoid their antibacterial or physiological effects. The experimental diets were introduced at weaning (d −6).

**Table 1:** Ingredient composition of experimental control diet (as-fed basis)

**Ingredient, %**	**Control diet**
Corn, ground	40.93
Dried whey	20.00
Soybean meal, 47%	10.00
Fishmeal	10.00
Lactose	7.22
Soy protein concentrate	5.00
Poultry byproduct meal	3.22
Soybean oil	2.92
Mineral premix^1^	0.35
Vitamin premix^2^	0.20
L-Lys HCl	0.06
DL-Met	0.05
L-Thr	0.03
L-Trp	0.02
Calculated energy and nutrient levels	
ME, kcal/kg	3,480
CP, %	22.53
Fat, %	6.48
Ca, %	0.80
P, %	0.73
Available P, %	0.51
Lys, %	1.50
Lactose, %	21.00

^1^Provided as milligrams per kilogram of diet: 3,000 of NaCl; 100 of Zn from zinc oxide; 90 of Fe from iron sulfate; 20 of Mn from manganese oxide; 8 of Cu from copper sulfate; 0.35 of I from calcium iodide; 0.30 of Se from sodium selenite.

^2^Provided per kilogram of diet: 2,273 μg of retinyl acetate; 17 μg of cholecalciferol; 88 mg of DL-α-tocopheryl acetate; 4 mg of menadione from menadione sodium bisulfite complex; 33 mg of niacin; 24 mg of D-Ca-pantothenate; 9 mg of riboflavin; 35 μg of vitamin B_12_; 324 mg of choline chloride.

### Feeding and sample collection

Pigs and feeders were weighed on the d of weaning (d −6), the d of the first inoculation (d 0), and d 5, for calculation of average daily gain (**ADG**), average daily feed intake (**ADFI**), and gain to feed ratio (**G:F**). Diarrhea score was assessed visually with a score from 1 to 5 (1 = normal feces, 2 = moist feces, 3 = mild diarrhea, 4 = severe diarrhea, and 5 = watery diarrhea) daily from d 0 by 1 scorer who was blind to the dietary treatments. Frequency of diarrhea was calculated by counting pig d with diarrhea score of 3 or higher.

The standard *E. coli* vaccine was withheld from the dams of the pigs used in this experiment, as were all routine treatments of the piglets with antibiotics. Prior to weaning, fecal samples of the sows from which we obtained the piglets for this experiment were collected to verify if they were negative for β-hemolytic coliforms by plating on blood and McConkey agars. Plates were incubated at 37°C and 5% CO_2_ for 24 h before reading. Populations of both total coliforms and β-hemolytic coliforms on blood agar were assessed visually. In the present study β-hemolytic coliforms were detected in the sow feces but they were not the pathogenic *E. coli* we used.

One-half of the pigs (16 from the challenged group (4 from each dietary treatment) and 16 from the sham group (4 from each dietary treatment)) were euthanized on d 5 post inoculation (**PI**) and the remainder on d 6 PI. Prior to euthanasia, pigs were anesthetized by intramuscular injection of a 1-mL combination of telazol, ketamine, and xylazine (2:1:1) per 23 kg of body weight. The final mixture contained 100 mg telazol, 50 mg ketamine, and 50 mg xylazine in 1 mL (Fort Dodge Animal Health, For Dodge, IA). After anesthesia, pigs were euthanized by intracardiac injection of 78 mg sodium pentobarbital per 1 kg of BW (Fort Dodge Animal Health, For Dodge, IA).

Mesenteric lymph nodes were aseptically collected then pooled within pig, ground, diluted and plated on brain heart infusion agar for measurement of total bacteria and the results were expressed as CFU per g of lymph node [19].

Three-cm samples of ileum and colon were collected and cut with scissors longitudinally in the mesenteric border. Tissues were gently washed in buffered saline then fixed in Carnoy’s solution for 2–3 h. Subsequently tissue samples were placed in 100% ethanol, 95% ethanol, and 70% ethanol for 30 min each and maintained in 70% ethanol until the staining process. The fixed intestinal tissues were embedded in paraffin, sectioned at 5 μm and stained with high iron diamine (**HID**) and alcian blue (**AB**), pH 2.5, as previously described [20].

### Sample processing and analysis

After staining, the slides were scanned by NanoZoomer Digital Pathology System (Hamamatsu Co., Bridgewater, NJ), and the measurements were conducted in NanoZoomer Digital Pathology Image Program (Hamamatsu Co., Bridgewater, NJ). Measurements included villus height, crypt depth, and the cross-sectional area of sulfo- (stained brown) and sialomucin (stained blue). The measurements for villus height and crypt depth were performed on 10 well-oriented villi [21] scanned at 40× resolution.

The total number of goblet cells per villus was counted and NDP.view software was used to measure the cross-sectional area (μm^2^) of individual goblet cells. The measurements were performed in 3 well-oriented villi scanned at 40x resolution.

### Statistical analysis

Data were subjected to an analysis of variance using the Proc Mixed procedure (SAS Inst. Inc., Cary, NC). Pig was the experimental unit. The statistical model included effects of *E. coli* challenge, diet, and their interaction as fixed effects and group as a random effect. Specific contrasts were used to test comparisons between the control and the clay treatments collectively within each challenge group. In addition, differences among the clay treatments within each challenge group were tested by pair-wise comparisons when the overall main effect or the diet x challenge interaction was significant. The χ^2^ test was used for the frequency of diarrhea. The α levels of 0.05 and between 0.05 and 0.10 were used for determination of significance and tendency, respectively, among means.

## Results and discussion

After the challenge, fecal samples were collected from pigs from sham and *E.coli*-challenged groups and it was observed that both groups of pigs carried β-hemolytic *E. coli*. Subsequent PCR analysis [22] showed that the sham-challenged pigs carried *E. coli* that produced cytotoxic necrotizing factor. This minor background infection with a wild strain of *E. coli* occurred in some of the sham-challenged and *E.coli*-challenged pigs in this experiment indicating that the sham-challenged pigs had pathogenic organisms and that the *E.coli*-challenged pigs could have other pathogenic organisms besides the challenge one, so the model represents a multiple infection rather than an uncomplicated single-pathogen challenge.

Cytotoxic necrotizing factor is produced by 40% of pathogenic *E. coli* strains involved in urinary tract infections and 5-30% of those involved in diarrheic infections [23]; it increases adherence of the pathogen to epithelial cells. The impact of infection with this wild strain on the response to the challenge strain is unclear, but if clays provide protection from diarrhea by strengthening the mucus barrier, they should provide similar protection from both of these strains of *E. coli*.

### Diarrhea score and growth performance

The *E. coli* challenge was successful as it increased diarrhea score moderately from d 3 to 5 (Table 2) and reduced ADG from d 0 to 5 PI (Table 3), consistent with previous results [8]. The diarrhea scores were low during the first d after challenge, apparently reflecting a lag period after the inoculation before the clinical signs appeared (Table 2). During this period the *E.coli*-challenged pigs actually had lower diarrhea scores (*P* < 0.05) than did the sham-challenged ones. During the active disease, from d 3 to 5 PI, the *E.coli*-challenged pigs had a higher diarrhea score than the sham-challenged pigs (*P* < 0.05), as expected (Table 3).

**Table 2:** **Effect of clays on diarrhea score of pigs experimentally infected with a pathogenic *E. coli***^**1**^

	**Treatment**^**2**^									***P*-value**				
	**Sham**				***E. coli***					**Main effect**^**3**^			**CON vs. Clays**^**4**^	
**Item**	**CON**	**SMA**	**SMB**	**ZEO**	**CON**	**SMA**	**SMB**	**ZEO**	**SEM**	***E. coli***	**Diet**	**E x D**	**Sham**	***E. coli***
d 0 to 2^5^	2.02	2.33	2.23	2.21	1.50	1.94	2.00	1.60	0.19	0.03	0.46	0.91	0.27	0.45
d 3 to 5	2.37	1.98	2.52	1.87	2.64	3.04	2.94	2.50	0.24	0.01	0.45	0.66	0.64	0.52
d 0 to 5	2.20	2.16	2.37	2.04	2.07	2.49	2.47	2.05	0.17	0.65	0.39	0.81	0.34	0.98
Pig d^6^	48	48	48	48	48	48	48	48	-	-	-	-	-	-
Diarrhea d^7^	3	4	4	4	3	8	8	5	-	-	-	-	-	-
Frequency, %^8^	6.25	8.33	8.33	8.33	6.25	16.67	16.67	10.42	-	0.13	0.14	0.08	0.64	0.13

^1^n = 8 pigs/treatment.

^2^Sham = unchallenged; *E. coli* = *E. coli* challenged; CON = control diet; SMA = 0.3% smectite A; SMB = 0.3% smectite B; ZEO = 0.3% zeolite.

^3^*E. coli* = *E. coli* challenge effect; Diet = diet effect; E x D = interaction between *E. coli* and diet effects.

^4^Contrast between CON and all clay treatments within challenge treatments.

^5^Diarrhea score = 1, normal feces, 2, moist feces, 3, mild diarrhea, 4, severe diarrhea, 5, watery diarrhea.

^6^Pig d = number of pigs x the number of d of diarrhea scoring.

^7^Diarrhea d = number of pig days with diarrhea score ≥ 3. Statistical analysis was conducted by chi-square test.

^8^Frequency (frequency of diarrhea during the entire experimental period) = diarrhea days*100/pig days.

**Table 3:** **Effect of clays on growth performance of pigs experimentally infected with a pathogenic *E. coli***^**1**^

	**Treatment**^**2**^									***P*-value**				
	**Sham**				***E. coli***					**Main effect**^**3**^			**CON vs. SM**^**4**^	
**Item**	**CON**	**SMA**	**SMB**	**ZEO**	**CON**	**SMA**	**SMB**	**ZEO**	**SEM**	***E. coli***	**Diet**	**E x D**	**Sham**	***E. coli***
**d −6 to 0**														
ADG, g	6.25	29.17	−2.08	−25.00	12.50	−25.00	33.33	8.33	42.4	0.80	0.86	0.38	0.87	0.84
ADFI, g	394	442	319	367	421	421	329	406	212	0.74	0.31	0.96	0.79	0.60
**d 0 to 5**														
ADG, g	237	180	157	187	137	132	122	85	63.71	< 0.01	0.52	0.73	0.16	0.58
ADFI, g	715	715	557	632	632	627	455	517	193	0.11	0.15	1.00	0.42	0.32
G:F^5^	0.34	0.26	0.33	0.32	0.23	0.24	0.24	0.24	0.048	0.11	0.95	0.92	0.64	0.95

^1^n = 8 pigs/treatment.

^2^Sham = unchallenged; *E. coli* = *E. coli* challenged; CON = control diet; SMA = 0.3% smectite A; SMB = 0.3% smectite B; ZEO = 0.3% zeolite.

^3^*E. coli* = *E. coli* challenge effect; Diet = diet effect; E x D = interaction between *E. coli* and diet effects.

^4^Contrast between CON and all clay treatments within challenge treatments.

^5^G:F was not reported for period −6 to 0 because of the negative values for ADG.

There were no dietary effects on either diarrhea scores (Table 2) or growth performance (Table 3), in contrast to the beneficial effects of clays on diarrhea score is in our earlier results [8]. Our earlier experiments [8] continued for 12 d after inoculation, well into the recovery phase. The pigs in the present experiment were euthanized at around the peak of disease (d 5 and 6 PI) in order to measure physiological effects of the *E. coli* challenge and the clays at that crucial time. Therefore, diarrhea was assessed for only a short time, with the critical period being d 3–5 PI. It is not clear if we would have observed the same effects on diarrhea score as we did earlier [8] if the experiment had been carried out until the recovery phase. In one of our earlier experiments clays reduced diarrhea during d 3–6 PI; whereas in the other there was only a trend during d 3–6 PI but clearer effects later [8]. The benefits of clays in reducing diarrhea that we reported [8] are supported by research in humans, as a meta-analysis of 9 studies showed that children with acute gastroenteritis consistently had lower duration of diarrhea when treated with smectite along with re-hydration compared with a placebo group without smectite [24].

### Goblet cell number and size

Goblet cells in the intestine produce mucins, the proteins that comprise the bulk of the mucus layer which acts as the first line of defense against enteric infections [25]. The present results show that the *E. coli* challenge increased both the number and size of goblet cells in the ileum (Table 4), consistent with an increase in mucin secretion in response to pathogenic bacteria or intestinal microbes that has been previously reported [21,26,27]. Perhaps the increased mucin production is a protective response. One of the clays (SMA) tended to increase goblet cell size in the ileum (*P* = 0.07) when compared to BAS in the *E.coli*-challenged group. There was a trend (*P* = 0.06) for an interaction between diet and challenge on ileal goblet cell number in which one clay (SMB) increased the number of goblet cells in challenged pigs only. There was a diet effect on goblet cell size in the colon (Table 4) in which the clays generally increased goblet cell size, mostly in the sham group. These modest increases in goblet cell size and number during the acute phase of the infection when clays were fed may reflect enhanced protection and may at least partially explain the reduction in diarrhea observed previously in pigs [8] and children [24].

**Table 4:** **Effect of clays on goblet cell number and size in ileum and colon of pigs experimentally infected with a pathogenic *E. coli***^**1**^

	**Treatment**^**2**^									***P*-value**				
	**Sham**				***E. coli***					**Main effect**^**3**^			**CON vs. SM**^**4**^	
**Item**	**CON**	**SMA**	**SMB**	**ZEO**	**CON**	**SMA**	**SMB**	**ZEO**	**SEM**	***E. coli***	**Diet**	**E x D**	**Sham**	***E. coli***
**Ileum**														
Number^5^	25.54	23.58	23.67	25.62	27.42	25.00	32.42	26.87	3.08	< 0.01	0.16	0.06	0.49	0.71
Size^6,7^, μm^2^	29.47^b^	29.58^b^	31.88^a,b^	30.72^b^	31.00^a,b^	35.96^a^	30.65^b^	31.89^a,b^	0.764	0.01	0.18	0.01	0.32	0.15
**Colon**														
Number	28.21	24.75	27.54	25.83	27.71	28.85	26.67	24.18	12.71	0.84	0.43	0.39	0.29	0.60
Size, μm^2^	26.26	27.33	27.60	30.37	25.56	28.31	25.82	27.69	0.801	0.20	0.04	0.41	0.09	0.21

^a-b^ Means with different superscripts in the same row differ.

^1^n = 8 pigs/treatment.

^2^Sham = unchallenged; *E. coli* = *E. coli* challenged; CON = control diet; SMA = 0.3% smectite A; SMB = 0.3% smectite B; ZEO = 0.3% zeolite.

^3^*E. coli* = *E. coli* challenge effect; Diet = diet effect; E x D = interaction between *E. coli* and diet effects.

^4^Contrast between CON and all clay treatments within challenge treatments.

^5^Goblet cell number; total number of goblet cells per villus, average of 3 villi.

^6^Goblet cell size, cross-sectional area.

^7^Con vs. SMA (Tukey adjustment) *P* = 0.07.

### Bacterial translocation

The *E. coli* challenge clearly increased bacterial translocation from the lumen to the lymph nodes but the dietary treatments did not detectably alter it (Table 5). To our knowledge, bacterial translocation from the intestinal lumen to the mesenteric lymph nodes has not been reported for pigs challenged with a pathogenic *E. coli* strain*.* Chicks infected with *Eimeria acervulina*, *E. maxima*, and *Clostridium perfringes* exhibited increased bacterial translocation from intestinal lumen to the spleen when compared with control birds [26] indicating that enteric infections reduce the integrity of the intestinal barrier. The increased total bacterial translocation caused by *E. coli* in the present study (Table 5) indicates that the infection reduced the effectiveness of the intestinal barrier, which was expected.

**Table 5:** **Effects of clays on bacteria in lymph nodes of pigs experimentally infected with a pathogenic *E. coli***^**1**^

	**Treatment**^**2**^									***P*-value**				
	**Sham**				***E. coli***					**Main effect**^**3**^			**CON vs. Clays**^**4**^	
**Item**	**CON**	**SMA**	**SMB**	**ZEO**	**CON**	**SMA**	**SMB**	**ZEO**	**SEM**	***E. coli***	**Diet**	**E x D**	**Sham**	***E. coli***
**Log**_**10 **_**CFU**^**5**^	1.05	0.74	0.65	0.60	1.87	2.12	2.03	1.69	0.30	0.01	0.88	0.90	0.44	0.87

^1^n = 64 (8 pigs/treatment).

^2^Sham = unchallenged; *E. coli* = *E. coli* challenged; CON = control diet; SMA = 0.3% smectite A; SMB = 0.3% smectite B; ZEO = 0.3% zeolite.

^3^*E. coli* = *E. coli* challenge effect; Diet = diet effect; E x D = interaction between *E. coli* and diet effects.

^4^Contrast between CON and all clay treatments within challenge treatments.

^5^Log_10_ CFU/g of lymph node.

### Intestinal morphology

Weaning triggers a reduction in villus height and in the villus height:crypt depth ratio, caused at least partially by interruption of voluntary feed intake [28], and restoration of villus height may be important for health and growth performance of the pig. In the present study, the challenge increased crypt depth and tended to reduce the villus height:crypt depth ratio (VH:CD; Table 6) as shown previously [17]. These effects of disease may exacerbate the detrimental impact of weaning on pig health and growth. The response to *E. coli* is inconsistent across experiments. Our observed values for the sham group are similar to previously reported in some cases [29] but smaller than those previously reported [17,30] in others. We did not detect any effect of clays or challenge on intestinal morphology (Table 6) except for a tendency (*P* = 0.07) for the effects of clays in increasing VH:CD in the *E.coli* challenged pigs. Beneficial effects of small amounts of dietary clays have been reported previously. For example, montmorillonite increased villus height and villus height: crypt depth ratio in jejunum when fed to weanling pigs at 0.15% of the diet [13]. Similar results were obtained in broiler chickens. Previous authors [14,30,31] reported that feeding 0.1%, or 0.2% montmorillonite increased villus height and reduced crypt depth in the duodenum and jejunum.

**Table 6:** **Effect of clays on intestinal morphology of pigs experimentally infected with a pathogenic *E. coli***^**1**^

	**Treatment**^**2**^									***P*-value**				
	**Sham**				***E. coli***					**Main effect**^**3**^			**CON vs. Clays**^**4**^	
**Item**	**CON**	**SMA**	**SMB**	**ZEO**	**CON**	**SMA**	**SMB**	**ZEO**	**SEM**	***E. coli***	**Diet**	**E x D**	**Sham**	***E. coli***
**Duodenum**														
VH^5^	384.6	374.6	380.6	359.1	356.6	393.8	382.5	365.0	21.06	0.99	0.78	0.80	0.64	0.40
CD^6^	264.9	276.3	263.8	262.0	273.5	257.1	259.9	275.45	39.15	0.98	0.95	0.63	0.87	0.55
VH:CD^7^	1.55	1.48	1.53	1.47	1.39	1.84	1.61	1.45	0.26	0.42	0.25	0.15	0.70	0.07
**Ileum**														
VH	299.0	310.7	288.7	305.8	305.4	289.8	282.1	309.9	9.15	0.64	0.36	0.72	0.85	0.45
CD	208.4	214.1	212.8	222.4	228.5	232.3	230.8	217.6	8.37	0.05	0.96	0.48	0.45	0.88
VH:CD	1.44	1.49	1.37	1.38	1.34	1.25	1.25	1.45	0.08	0.10	0.61	0.35	0.76	0.81
**Colon**														
CD	236.0	229.0	247. 7	227.5	228.8	244.2	223.1	216.4	73.94	0.28	0.36	0.18	0.90	0.93

^1^n = 8 pigs/treatment.

^2^Sham = unchallenged; *E. coli* = *E. coli* challenged; CON = control diet; SMA = 0.3% smectite A; SMB = 0.3% smectite B; ZEO = 0.3% zeolite.

^3^*E. coli* = *E. coli* challenge effect; Diet = diet effect; E x D = interaction between *E. coli* and diet effects.

^4^Contrast between CON and all clay treatments within challenge treatments.

^5^Villus height, μm.

^6^Crypt depth, μm.

^7^Villus height:crypt depth ratio.

### Sulfo- and sialomucin

Mucins can be acidic or neutral. Acidic mucins are comprised of sulfo- and sialomucins. The body often reacts to infection by increasing the secretion of sulfomucins [32] as a protective mechanism; the present data do not show that response (Table 7). The present results do not show effects of either infection or dietary clays on the relative amount of sulfo- and sialomucins within goblet cells (Table 7).

**Table 7:** **Effect of clays on relative amounts of sulfo- and sialomucin area of pigs experimentally infected with a pathogenic *E. coli***^**1**^

	**Treatment**^**2**^									***P*-value**				
	**Sham**				***E. coli***					**Main effect**^**3**^			**CON vs. Clays**^**4**^	
**Item**	**CON**	**SMA**	**SMB**	**ZEO**	**CON**	**SMA**	**SMB**	**ZEO**	**SEM**	***E. coli***	**Diet**	**E x D**	**Sham**	***E. coli***
**Ileum**														
Sulfo^5^	44.31	37.59	32.86	37.95	31.28	32.31	37.38	37.10	5.80	0.52	0.97	0.73	0.37	0.65
Sialo^6^	55.69	62.41	67.14	62.05	68.62	67.69	62.62	62.90	5.80	0.52	0.97	0.73	0.37	0.65
**Colon**														
Sulfo	92.39	92.74	95.09	94.96	93.37	90.49	87.60	94.29	1.57	0.14	0.43	0.26	0.45	0.33
Sialo	7.61	7.26	4.91	5.03	6.63	9.51	12.40	5.71	1.57	0.14	0.43	0.26	0.45	0.33

^1^n = 8 pigs/treatment.

^2^Sham = unchallenged; *E. coli* = *E. coli* challenged; CON = control diet; SMA = 0.3% smectite A; SMB = 0.3% smectite B; ZEO = 0.3% zeolite.

^3^*E. coli* = *E. coli* challenge effect; Diet = diet effect; E x D = interaction between *E. coli* and diet effects.

^4^Contrast between CON and all clay treatments within challenge treatments.

^5^Sulfo = % of total sulfo- and sialomucin area that is sulfamucin.

^6^Sialo = % of total sulfo- and sialomucin area that is sialomucin.

## Conclusions

The present results provide novel information regarding the physiological responses in the intestinal barrier of pigs to a challenge with a pathogenic *E. coli* strain. To our knowledge, it is the first time that bacterial translocation from intestinal lumen to mesenteric lymph nodes and goblet cell size and number in weaned pigs challenged with a pathogenic *E. coli* is reported. Both the infection and SMA altered goblet cell size and number. The clinical benefits of clays in the face of enteric infections that we observed in previous experiments with pigs, such as the reduction in diarrhea score, did not occur in this shorter experiment, but it is unclear whether they may have appeared if the experiment had been longer. However, it was important to explore the potential beneficial of the clays during the acute phase of an enteric infection.

## Abbreviations

PI: Post inoculation; SMA: Smectite A; SMB: Smectite B; ZEO: Zeolite; ETEC: Enterotoxigenic; HID: High iron diamine; AB: Alcian blue.

## Competing interests

Dr. Orlando Osuna, is employed by Milwhite, a company that manufactures and markets clays.

## Authors’ contributions

JASA carried out the animal work, processed the samples, participated in the design of the study, performed the statistical analysis, and drafted the manuscript. YL carried out the lymph node assay and participated in the design of the study. MS participated in the design of the study and performed the training for diarrhea score assessment. JJL helped with the animal work, and carried out the goblet cell size and number quantification. HRG participated in the design of the experiment. CWM provided the *E. coli* for the challenge, and participated in the design of the experiment. OO participated in the design of the experiment. JEP participated in the design of the experiment and helped to draft the manuscript. All authors read and approved the final manuscript.
